# U.S. REMOTE CANCER CARE FOR OLDER ADULTS: A SYSTEMATIC REVIEW AND SOCIAL NETWORK ANALYSIS

**DOI:** 10.1093/geroni/igad104.3677

**Published:** 2023-12-21

**Authors:** Edwin Garces, Maki Karakida

**Affiliations:** Portland State University, Portland, Oregon, United States; University of Massachusetts, Allston, Massachusetts, United States

## Abstract

Telemedicine and telehealth services are a promising solution for older cancer patients who are susceptible to infectious diseases, like COVID-19, and travel a longer distance to their closest hospital. To provide all-inclusive oncology care, understanding social disparities in access to remote cancer care is crucial. Yet, little is known about the accessibility of these technologies among US cancer patients in later life. The present study aimed to evaluate the main sociocultural factors associated with older patients of virtual oncology care interventions in the US. This systematic review explored the literature on telemedicine for geriatric oncology and patients’ accessibility to remote health technologies. PubMed, CINAHL, Scopus, and Web of Science databases were searched. Using Bibliometrix, Science of Science (Sci2) Tool, and Gephi, Social Network Analysis (SNA) was conducted to identify the central and intermediate factors linked to remote cancer care for older US adults. Among 427 retrieved articles, 19 articles, published between 2003 and 2023, met the eligibility criteria. The selected studies were focused on rural cancer patients (20%) and telehealth during COVID-19 outbreaks (20%), followed by lifestyle interventions (15%), ehealth literacy (10%), prostate or breast cancer (5%), older sexual minority men with prostate cancer (5%), and patients’ race, age, and region (5%). Findings also highlighted that gender, digital health literacy (technology readiness), and location are the key determinants of telemedicine access and use among older US cancer patients. Multiple socio-demographic aspects of older patients need to be considered for implementing equitable access to remote oncology care.

